# Weaving birth: interdependence and the fungal turn

**DOI:** 10.3389/fgwh.2025.1642537

**Published:** 2025-09-10

**Authors:** Michelle Sadler, Sara Cohen Shabot

**Affiliations:** 1 Departamento de Historia y Ciencias Sociales, Facultad de Artes Liberales, Universidad Adolfo Ibáñez, Santiago, Chile; 2 The Women's and Gender Studies Program, The Faculty of Humanities, University of Haifa, Mount Carmel, Israel

**Keywords:** fungal turn, interconnectedness, interdependence, phenomenology, positive childbirth

## Abstract

In this article, we approach childbirth through the lens of the “fungal turn,” using fungal mycelial networks as a conceptual and metaphorical resource for rethinking birth as a relational experience of collective care. Like fungi, which thrive through mutualistic, multispecies relationships, childbirth unfolds within dense networks of biological, social, and ecological connections; between pregnant person and fetus, caregivers, communities, and environments. We draw on our own contrasting childbirth experiences -one shaped by obstetric violence and the need for hyper-vigilant control, the other by trust, safety, and the capacity to surrender- to illustrate how different models of care either reinforce the logic of autonomous, isolated, and bounded birthing subjects or, in contrast, highlight their vulnerability, interconnectedness, and permeability. Our analysis combines a descriptive phenomenological approach, to convey the lived experience of birth in its sensory, embodied immediacy, with a hermeneutical phenomenological approach, which situates and interprets these experiences within the broader cultural and relational frameworks that shape them. Phenomenological insights on intercorporeality challenge the idea of the autonomous subject, reframing subjectivity as emerging through inherently embodied and interconnected engagements with others and the world. In this framework, the fungal metaphor illuminates how the weaving of interdependence unsettles dominant modern conceptions of agency and individuation, offering new ways to imagine what constitutes a positive birth.

Everything is about weaving. To weave is to understand interdependence; it is to grasp reciprocity, the constant and ongoing interaction between all phenomena. So, weaving is not just a physical act—it's a metaphor. The real weaving is what species do, what symbiotic forms do, what mycelial forms do. [Vicuña C (1)]

## Introduction

Testimonies of birth experiences described as positive, as fostering a sense of well-being in the birthing subject, and even as empowering, share some common features. In a nutshell, these are experiences in which birthing women felt supported, safe, respected, and in control (though, as we shall see, “control” can look very different across contexts): experiences in which they participated in the decisions made during the process and felt like autonomous agents throughout (2, 3)^1^. In these experiences (which are not necessarily the same as those that lead to “positive outcomes”—in the form of live births of “healthy babies” born to “healthy mothers”), the emphasis is on the compassionate treatment and respectful care with which the inherent vulnerability of the birthing subject is met (2, 7–9). Such respectful care sensibly sees and fosters the birthing woman's need to relax and open the body's boundaries, and paradoxically “lose” control (in the sense of rational planning).

What constitutes a positive birth experience varies greatly depending on context. Research shows that when birthing women cannot trust their care environment to prioritize their well-being, autonomy, and active participation in decisions throughout the birth will be key to her experience of the birth as successful and positive (10, 11). Conversely, when the birthing environment is experienced as safe and attuned to needs from the start, a “good” birth is often characterized by the ability to surrender control and allow the process to unfold with support (3). Indeed, and especially in the case of physiological and unmedicated births, recent literature refers to this state as “birthing consciousness”—a state often described as infused with transcendence, profound transformation, and creative energy, akin to what is experienced during certain altered states of consciousness (12–14).

In this paper, we bring together descriptive phenomenology**,** to convey the embodied immediacy of childbirth, and hermeneutical phenomenology**,** to interpret these experiences within broader contexts. We use this combined approach to explore recent research on the fungal turn and its potential to illuminate birthing contexts where the subject experiences itself as intertwined both with its surroundings and with the events unfolding within the body. The birthing body in a state of flow resembles the recent descriptions of a fungus: a form of being that defies hierarchies and traditional limits, that straddles life and death, organic and inorganic, plant and animal, singular and plural, and whose porosity and interconnection challenge rationality and autonomy (15–17).

We begin by sharing fragments of our own birthing experiences to ground the discussion in lived realities and to highlight how care practices shape birthing subjectivities. We then reflect on different models of childbirth care, examining how they either reinforce or challenge the notion of the autonomous, bounded birthing subject. Next, we draw on phenomenological insights about embodiment and bodily porosity to provide a theoretical foundation that prepares the way for our central engagement with the fungal turn as a metaphor and conceptual resource for rethinking birth as an entangled, relational process of collective care. Finally, we discuss how this framework reimagines positive birth experiences as ones that move beyond control and individualism toward connection and interdependence, where birth does not need to be “controlled,” rationally planned, or defended from unwanted intrusion, allowing the birthing person to safely become a “birthing being,” a “fungus” with open boundaries that intertwine and weave with the baby, the world, and others.

## Different contexts, changing needs: looking back at our birth experiences

The authors of this text have both written extensively about childbirth, each having given birth twice and having lived experiences that can be placed at both ends of the care continuum.

Sara Cohen Shabot (18) began writing about the topic after her second child was born:

A labor with apparently optimal results: no physical damage, healthy mother, healthy baby. Nothing to complain about; nothing to mourn. Nevertheless, this labor experience still haunts me and has informed almost all of my academic writing since that time. Today I can say truthfully that I suffered from obstetric violence and that, in more ways than one, this was a traumatic experience. (232)

Sara's birthing experience was marked by feelings of deep abandonment, of loss of autonomy, and of lack of care. She arrived at the hospital at 8 cm of dilation and was then connected to a fetal monitor and left sitting there, unable to move freely while she experienced intense contractions. This went on for nearly five hours, with minimal interactions other than a midwife arriving periodically to perform vaginal examinations and saying “You’re not progressing” after each one of them. After several such exams, Sara refused to undergo another one. The midwife then went to find an obstetrician, who came in to reprimand Sara for resisting and not “cooperating.” But although the obstetrician then threatened a cesarean section, Sara was able to achieve a vaginal birth thanks to the intervention of another midwife, who asked the obstetrician not to proceed with surgery without one last examination, which confirmed that the birth was imminent. Throughout her experience, Sara felt profoundly abandoned and mistreated. The few moments that she remembers as “good” were those in which she was able, with the support of her partner and/or the second midwife, to defend herself and assert her sovereignty, by resisting further interventions and avoiding unnecessary procedures, including the cesarean section that had been about to be performed. She felt that she had to be constantly on alert and exercising control, defending her needs and desires to whatever extent she could.

Michelle Sadler, on the other hand, began researching childbirth a decade before ever becoming a mother, as an anthropology student conducting fieldwork in public maternity hospitals in Chile. Her experience as a researcher and activist equipped her with the tools to navigate the healthcare system in such a way that her birthing experience, when the time came, was one of comprehensive care. She found healthcare professionals and institutions that supported her desire for physiological, unmedicated births, allowing her to let go of control and fully immerse herself in the experience with confidence. For both of her childbirth experiences, she has a fairly clear recollection of events up until the final few hours of labor. For the last two or three hours, however, while she can provide a highly detailed account of how she herself was feeling in terms of her embodied experience—in some ways even more so than for the earlier stages—she has almost no recollection of what was happening around her. She doesn't remember how many people were in the room or what they were doing (unless they were right beside her), and she can't visualize the physical surroundings. In those final moments, her sense was that everything around her became blurry, even the sounds, and she felt a lack of clear boundaries, as if she were connected to everything in a way that is hard to articulate, perhaps even transcendental. She recalls following the midwives' suggestions and actions to support the labor process. In her first labor, they guided and supported her while she did squats, attempting to help progress in a labor that had already lasted more than 30 h. In her second labor, they recommended a hot shower and supported her in the water as well as later, as she moved to the floor, onto a hands-and-knees position. Only after the birth did she look “outward” again and realize what the environment around her was like: She was surprised to see several people who had not been present earlier and to notice the medical equipment that had appeared in the room. For Michelle, the experience most similar to the intense moments of childbirth was the altered states of consciousness she had encountered in her early twenties through breathing exercises and meditation, after several years of training with Mexican tutors who had, in turn, learned from shamans. Although that earlier experience was far less physically intense, she found the sense of interconnectedness, trust, and being cared for strikingly similar. In both experiences, it was crucial to have caretakers who could support and contain her throughout the journey. So, in Michelle's birthing experiences, what stands out most are feelings of fluidity, yielding, and trust—all of which are at odds with control and decision-making.

In both Sara's and Michelle's birth experiences, no matter how different they were, their most basic expectation and need was to be cared for in a way that allowed them to navigate and go through the birthing journey. And this is what most women and birthing subjects report needing during childbirth. The meta-synthesis carried out by Downe et al. (19), of studies that look into what matters to women for labor and birth, reports that women

recognized the potential vulnerability of themselves and their baby through the process, and the essential uncertainty about what might happen. This was associated with a strong desire for safe, supportive, kind, respectful and responsive care during labor and birth. These characteristics applied to birth companions, professional and lay care givers, and to the processes and environment of care. (12)

Unfortunately, these needs are not always met. As demonstrated by the extensive evidence of worldwide obstetric abuse, disrespect, mistreatment, and violence, birthing women are often ignored and neglected and their childbirth experiences, as a result, are often negative and even traumatic (20–25).

Because they are aware of this ahead of time, many women approach the healthcare system with suspicion, feeling the need to control whatever dimensions they can in order to have an experience that aligns with their expectations. In many cases, they arrive with a body that has already been “domesticated,” silenced to meet the system's needs more than their own (26). Studies that focus on the expectations of women with previous birth experiences show an exacerbated need to exert control. A recent Australian study found that more than 85% of women would make different decisions for future births, “feeling they needed to strongly exert control, choices, and advocate for themselves in future” [ (11), 8]. A troubling issue that emerged was that many women felt guilty for not having been better informed, which fostered a desire for a different experience. This sense of self-blame, tied to failures within the system, only intensified the trauma they experienced, reinforcing the conclusions found in other studies (27, 28). These feelings were even more pronounced when previous birth experiences had been negative or traumatic, as the women often had a strong urge to avoid repeating those experiences and to feel in control of their birth choices (10).

Thus, the idea of control takes on a variety of very different shapes. In Sara's birth experience, she was never able to feel that she could release control, given the threatening environment in which she was going through labor—and in that context, she experienced being in as much control as possible as the safest part of the birthing experience. Michelle's experience, on the other hand, involved being in control of managing external factors and decisions (about where and with whom to birth) *previous* to beginning labor. This meant that once she was in labor, she could let go and follow the flow of her needs, while feeling completely supported. As several authors have noted, this apparent paradox -being able to relinquish control and still feel fully in control- is a desirable part of the childbirth journey. In order for a woman to be able to surrender control during childbirth, she must first feel safe and in control of the process (3). Such a possibility is central to many positive childbirth experiences.

It is important to clarify that these testimonies represent the authors' personal birth experiences, offering insight into specific moments along the birthing continuum, whilst recognizing that birthing experiences are diverse and multifaceted, encompassing a wide range of contexts and emotions. The intention in sharing these stories is not to universalize them, but to use them as a foundation for exploring broader themes of control, vulnerability, and interconnectedness that resonate across many birthing experiences.^2^

## From separation to interconnectedness

Sara and Michelle's opposed birthing experiences reflect conflicting models of care and concepts of personhood. At one end of the spectrum are highly medicalized births, often involving a cascade of obstetric interventions within a hierarchical system of care in which medical professionals are regarded as the holders of authoritative knowledge (29). In this model, the “patient” is reduced to a physiological body responding to mechanical rules, with psychosocial factors being frequently neglected (3, 30, 31). Crucially, the birthing body is not just the body of any patient or person; it is a female body. This distinction is of utmost importance, because the biomedical model of childbirth reflects asymmetrical gender power dynamics in which female bodies are objectified. At the dawn of obstetrics, male physiognomy and physiology were considered the standard, which led to the portrayal of the female body and its processes as abnormalities or deviations in need of control (22, 32). As Villarmea (33) notes, Enlightenment-era medicine and philosophy supported the notion that the condition of women was to be inherently deficient, weak, and sick because it was governed by the reproductive function. Women's capacity for pregnancy led to the view that women's bodies were incapable of achieving full self-control, which was considered the standard of rationality. Thus, the undisciplined reproductive female body needed to be controlled and domesticated by disciplinary technologies that would re-feminize and re-objectify it (18, 34, 35).

Modern obstetrics expanded in parallel with the global spread of colonial, and later industrial, logics. Core principles such as the optimization of time, assembly-line production, and the systematic alienation and isolation of relational ties were increasingly imposed on childbirth. The result is the image of the technocratic model of childbirth (32, 36, 37) in its fullest expression: that of a childbearing woman left alone, strapped to a stretcher, and denied the ability to walk, eat, drink, or receive comfort and care from her loved ones. In many cases, she has been subjected to a series of routine obstetric interventions decided by others, with little or no space to voice her needs or wishes. Birth, once a site of connection, transformation, and community, thus becomes instead a scene of alienation and technocratic efficiency—echoing the same colonial and industrial ideologies that have long sought to control both land and bodies.

One powerful mechanism for objectifying female bodies is through individualization, isolation, and separation, as evoked in the previous paragraph. Biomedicine and obstetrics are profoundly shaped by the idea that we are discrete individuals —separated both from other people and from the environment. Despite challenges from research across multiple disciplines, this idea persists in popular culture and medical practice. A striking example is the dominant representation of pregnancy in contemporary Western culture, which casts the woman or birthing subject as a container and the fetus as its separate content (38, 39). Kingma (40) terms this the “fetal container model,” in which the fetus is regarded as independently growing within the mother. This model reduces pregnant persons to mere containers, paradoxically obscuring both the fetus's location within and its connection to them. Underlying this logic is the long-standing notion that women are governed by their reproductive capacity and are thus inherently irrational, which enables their transformation into such corporeal containers:

The move from a subject to a container concerns the deletion/removal/vanishing of that constitutive part of subjectivity that is reason. For, once we deprive a subject of her rationality (full capacity), it is easy to slide into treating it as an object—in this case, as a container. [(33), 74]

Such a view lays the ground for the maternal-fetal conflict in bioethics, which frames the fetus as a threat to the mother and vice versa, in a dynamic that perpetuates the discursive separation between the two (41). Multiple scientific fields include theories that share the common trope of the antagonistic relationship between the mother and the fetus, mirroring the self/other dichotomy in Cartesian dualism (42).

This kind of logic contradicts the understanding of pregnancy and childbirth as processes of cooperation and interconnectedness, an understanding that lies at the heart of humanistic and holistic models of care (31). These models advocate for a comprehensive, woman-centered approach to pregnancy and childbirth in which the psychosocial dimensions of care are central. Such a view fosters an environment in which the woman or birthing subject feels supported, encouraging a compassionate approach to maternal care. Here interconnectedness, not separation, prevails as the fundamental principle of care.

This understanding also challenges the dominant Western scientific notion of the pregnant subject and fetus as separate entities with clearly defined boundaries. Research on the physiology of pregnancy supports a model of association rather than conflict between the pregnant person and the fetus. This relationship is marked by deep physiological interdependence, demonstrated by the exchange of DNA, oxygen, nutrients, and immune cells through the placenta as well as by maternal-fetal microchimerism, in which maternal cells migrate into fetal tissues during pregnancy and breastfeeding (43, 44). The microbial colonization *in utero* during pregnancy and microbiome transmission during vaginal birth are inherently relational acts, in which bodily boundaries blur; the birth canal, skin-to-skin contact, and breastfeeding are all moments of microbial seeding—a transference of life and immunity (42, 45). Research suggests that birth can be described not as the emergence of a discrete individual (the baby), but as the formation of a new community (46–48). Birth, in this view, represents a transition from one set of symbiotic relationships to another, “for not only is the eukaryotic body being reproduced, but so also are the bodies of its symbiotic microbes and so is the set of relationships between these organic components” (47). Accordingly, the pregnant body may be reconfigured such that the material distinction between “mother” and “fetus” dissolves. Pregnancy can then be understood not merely as a bidirectional exchange but as part of a broader, integrated circulation of matter within a symbiotic system (42).

This integrated, relational view of pregnancy, in which maternal and fetal bodies are deeply interdependent and boundaries are fluid, provides a framework for understanding not only the physiological but also the experiential dimensions of childbirth. At a transcendent level of connectedness, we can recall Michelle's birthing experiences, in which she felt secure and free enough to surrender to the experience she was living, in a way that was similar to what she had felt during altered states of consciousness through meditation. Such a retreat or withdrawal “into an inner world where time seemed to be suspended” [(3), 4] is a common experience reported by women when experiencing unmedicated physiological births in which they feel safe (3, 12). Dahan has proposed the concept of “birthing consciousness” to refer to this withdrawal into an inner world, which allows women to focus on the laboring process and facilitates the feeling that they can cope. The experience of childbirth is a transformative event that can deeply affect a woman's perception of reality, self, and the world around her. This psycho-physical altered state is often likened to other mystical or transcendent experiences, in which ordinary perceptions and boundaries of the self are expanded or redefined (12, 13). During altered states of consciousness, as reported in the literature, there is an enhanced feeling of interconnectedness in which the person feels at one with their surroundings, accompanied by a feeling of being protected and of being more than oneself (49).

In the following, we explore how phenomenological analyses that emphasize intercorporeality and that discuss interconnectedness and intertwining as the essential conditions of life itself might shed light on how a protected birth that allows for blurring boundaries and porous encounters can result in such a rewarding and positive experience. Later, we will use the metaphor of fungi to further explore these issues.

## Phenomenology and intercorporeality

Phenomenology challenges the idea that individuals exist as isolated atoms in the world (50, 51). Ever since Merleau-Ponty's discussions of the “body proper” or “one's own body” (*le corps propre*) as always already “cracked,” open, and intertwined with the world, and thus a precursor of intercorporeality, phenomenological analyses have emphasized that the clear separation and distinction between the subject and its world is nothing more than an analytical tool intended to provide us with the illusion of well-organized, methodical, “straight” thinking. The point of this illusory thinking is to allow us to detach ourselves from the world and examine it “from the outside,” so to speak, when in fact reality is messy and ambiguous and cannot be fully grasped through distinction, detachment, and separation. We are in the world, haunting space and haunted by it, profoundly linked to each other through material intercorporeality:

The concept of intercorporeality is thus deeply ambiguous: on the one hand it suggests continuity between myself and the other, an absence of definite boundaries, but this continuity is made possible only because of a sense of *discontinuity*, estrangement, anonymity, even dispossession, that prevents my body from ever being unambiguously my own. [ (52), 198; emphasis in the original]

This conceptualization of one's own body or the body proper as always already discontinuous in itself, always strange to itself, can be seen as clearly illustrating and supporting the understanding of the pregnant and birthing body discussed earlier: as ambiguous, simultaneously singular and plural, intertwined with its insides, and cracked or fragmented in itself—and, for that reason, not the same as a simple recipient or object that is separate from the fetus that is growing within it.

Critical phenomenological analyses dealing with disease and disability are also pertinent here. Foth and Leibing (53), in their account of the “being with dementia,” follow these central phenomenological insights on embodiment and intercorporeality to challenge the concept of the “person” as an isolated embodied entity and, with it, the “person-centered” approach to care: “Actually, the body is dependent on other bodies. … Thus, it is not possible to speak about the body as independent and distinct from other bodies. Only relations to other bodies and a liveable environment make bodily life possible and enable bodies to act” (5).

Phenomenological analyses of the ways in which the COVID pandemic revealed fundamental features of the human condition show how the requirements for isolation, for an exaggerated protection from the environment and others, in fact revealed our original interdependence and interconnectedness, which usually go unnoticed because they have been so deeply normalized and unquestioned: We are profoundly linked to others through our bodies, our fluids, our breaths, and our touch. Discussing the lessons of the pandemic in its aftermath, Butler (54) writes:

The definitive boundaries of the body presumed by most forms of individualism have been called into question as the invariable porosity of the body—its openings, its mucosal linings, its windpipes—all become salient matters of life and death. How, then, do we rethink bodily relations of interdependency, intertwinement, and porosity during these times? Or, rather, how do these times and this world, already shifting in intensity, offer a chance to reflect upon interdependency, intertwinement, and porosity? (33–34)

In other words, porosity, intertwinement, and interdependency are always already there; they characterize our way of being, our situation in the world and toward others. Merleau-Ponty's philosophy is perhaps the one that most clearly and meticulously discusses our intertwined condition, and Butler uses it to show how we are infused with the world:

The spatial limits of the perceived body belie its proper reach, for it is always both here and there, rooted and transported. The world that is usually assumed to be over there, or around me, is in fact already in and on me, and there is no easy way around that form of adherence, the way the world sticks to me and saturates me. (35)

Sara (4) has already employed these phenomenological insights to write about birth and breastfeeding, using the feminist conceptualization of “relational autonomy” and Beauvoir's ideas about the authentic subject as necessarily ambiguous and embodied, linked to others and to the world, and simultaneously immanent and transcendent, as tools for arguing that obstetric violence might be more accurately described as an injurious abandonment and a damage to the birthing subject's connections with others who are present at the birth, rather than primarily an offense to autonomy. Similarly, in her chapter on breastfeeding (55), she describes breastfeeding foremost as a practice that fosters connection and intercorporeality, precisely through the blurring of boundaries:

Breastfeeding [is] a fleshed experience through which we experience a “compelled generosity” and a basic intertwinement: between ourselves and the breastfeeding baby, between ourselves and the food we consume, the air we breathe, and the water we drink. The world enters us, nourishes us, and makes us in turn into nourishing bodies ourselves. From us, the world returns again to the outside, now as sticky, smelly, nurturing milk. The breastfeeding body, thus, appears here as an open body, an embodied, leaky, porous subjectivity entangled in the world. … We are giving and providing, but before that we have previously been nourished ourselves; we are the eating-edible body, meeting the world by going out to the world and receiving the world back into our embodied selves. We are “impure beings,” contaminating and contaminated, since our flesh is not really ours but an organic part of the organic world. (162, 165)

More recent, “posthuman,” accounts have followed this same path, resisting the conceptualization of embodiment as a solid substance and emphasizing instead the “watery” features of bodies. Neimanis (56) uses “bodies of water” as a feminist figuration with environmental, biological, and even ontological implications. Our bodies are in fact mostly made of water, a provocative fact that allows us to imagine our presence in the world, and the relations that we form and are part of, in different terms from those commonly adopted by neoliberal discourses:

“Bodies of water” trouble the idea of bodies as discrete and coherent individual subjects. As bodies of water we leak and seethe, our borders always vulnerable to rupture and renegotiation. As we know, our human bodies are at least two-thirds watery, but more importantly, these waters are in a constant process of intake, transformation, and exchange. For humans, the flush of waters sustains our bodies, but also connects them to other bodies and other environments—drinking, urinating, sweating, transfusing, siphoning, sponging, weeping. Human bodies are thus very literally implicated in other animal, vegetable and planetary bodies that materially flow through us, replenish us, and draw upon our own bodies as wells. This circulation inaugurates us into complex relations of gift, theft, and debt with all other life. (55)

In the following, we show how recent literature on fungi and the mycotic existence confirms and reinforces these phenomenological and posthuman approaches, emphasizing life as an expression of interconnectedness, porosity, fluidity, blurring of limits, and constant weaving.

## The fungal turn

There is a recent interest in fungi within the humanities: in what fungi and their position in the world may suggest about consensual ideas on the contours of subjects and objects and on the possibility of our existence as autonomous, individual, self-determining entities. Fungi belong to a distinct biological kingdom, defined by their unique structure: vast, branching networks of threads known as mycelia. These networks act as an ecological connective tissue, forming intricate webs that link organisms and environments in dynamic, evolving relationships. Unlike organisms that have discrete, isolated bodies, fungi grow as interconnected systems, merging and fusing in ways that challenge conventional notions of individuality. Through these networks, fungi not only sustain ecosystems but also embody the fundamental ecological principle of interdependence—reminding us that all life is enmeshed in mutual influence and exchange (16, 57). Ubiquitous and often unseen, fungi are in and around us, forming symbiotic partnerships with plants, animals, and even within human systems—offering nourishment and being nourished in return. As Alison Sperling notes [in (15)], the fungus serves as a “kind of model organism for ecological thinking—the mushroom not as an individual organism, but as always, a vast network underground, feeding and communicating with countless diverse species of plant and animal. It is a lifeform that animates new forms of thought and worlds with new (weirder) relations” (9).

The fungal turn is, thus, a specific kind of posthuman understanding of reality that anchors itself in the fungal in order to understand reality differently, for instance by challenging neoliberal and ableist models that privilege separateness, independence, and sovereignty and by generating new models that not only do not consider human beings as being superior to other forms of existence but also view them not as atomic and self-contained but rather as intertwined, permanently porous, and always in relationship. Fungi, thus, might help us to imagine a different kind of embodiment, one that resists clear limits and boundaries and challenges stability and solidity. In a special issue of the journal *Interconnections* devoted to the fungal turn, Mackey and Sendur (15) discuss how new media and diverse cultural products have adopted the fungus to create counter-discourses within, for instance, politics, ethics, and ontology:

Fungal discourses [are being used] to think about the porous and permeable limits of bodies, to reconsider our relationship with space, time, death and decay, and to imagine novel ways of perceiving, living, and resisting power. At this juncture, the fungal appears as a key concept which enables us to think within and through the many lines of flight presented to us by posthumanist thought: for example, environmental posthumanism, which rejects human exceptionalism and views entanglement as a legitimate form of rethinking our relations with others (human and non-), materializes in the constitutively relational nature of mycorrhizal networks, a process that threatens to dethrone any self-contained, rationally driven understanding of *Anthropos*. … The fungal opens up a space that new ontologies were seeking for a long time: a material, tangible ontology that cannot be accounted for with old forms of agency, individuation, form and matter. This is a posthuman ontology that embraces things in their becoming … that allows us to rethink relationality in its more intricate forms. (5)

In this same special issue, Victoria Jara (58) presents a provocative discussion of care based on insights deriving from the fungus as a figuration that resists conservative, capitalist, and colonialist models of care. As part of her analysis of the Mexican novel *Brujas*, she writes:

The collaborative work in the behaviour of fungi is mirrored in the character's web of care. … I propose that the similarity in the way both the chamana and fungi interpret their environments to establish relationships suggests that Feliciana is a fungal being in the sense that she is a regenerator, recycler, and networker that stitches the world together. She can see and cure illnesses, and fungi are key in that process. (85)

Can birth-carers, too, in other words midwives, partners, doulas, doctors, and friends and family, constitute “fungal beings” -“stitching the world together,” allowing and fostering this kind of blurring of boundaries for the birthing body, its insides, and the world surrounding it? We want to suggest that birthing in a fungal mode might facilitate the kind of positive, flowing, interconnected birth that we discussed above, a birth in which the mere prevention of trauma -through control, “optimal functionality,” and the possibility of repelling or resisting undesirable external interventions- is definitely not enough.

## Thinking birth through fungi

Fungi can serve as a compelling new metaphorical figuration for thinking about life—and about birth. The fungal turn (15–17) can offer powerful insights into childbirth as an entangled and relational experience of collective care, from which new communities emerge. We suggest that this metaphor is productively suggestive regarding what constitutes a good birth, where the weaving of interdependence unsettles and reconfigures dominant modern conceptions of agency, individuation, form, and matter.

Just as fungi thrive within interconnected mycelial networks, childbirth, too, unfolds within a web of external and internal relationships. Far from being isolated beings, the birthing subject and baby are deeply enmeshed with their environments, communities, and physiological processes. At one level, this entanglement is evident in the collective support systems that shape the birthing experience: midwives, doulas, family, and broader communities. The mycelium, which sustains ecosystems through connection and reciprocity, exemplifies this logic of interdependence and collaboration. And much as mycelial networks sustain and nourish life in the forest, these human networks foster care, safety, and empowerment during childbirth. On another level, entanglement occurs inwardly, in the rich exchanges between the pregnant person and the fetus. All of these interactions defy the notion of the gestating body as a passive container, highlighting instead a dynamic relationship marked by biological generosity, codependence, and mutual influence. This perspective reframes pregnancy not as the hosting of another life but as a process of shared becoming within a deeply entangled biological and relational ecology, which allows us to reimagine pregnancy and childbirth as a multispecies relationship. In this sense, such entanglement clearly challenges dualistic ontologies, such as the Cartesian one, where divisions between subjects and objects, mind and matter, are evident and definitive. By contrast, the ontology that emerges here emphasizes blurred boundaries and the absence of clear separations between the subject and its world. In line with Merleau-Ponty's ontology of the “flesh” (59), the ontology suggested by this “fungal turn” privileges intimate, carnal intertwining; and rejects purely dichotomous or abstract understandings of Being.

Fungi thrive on decay and decomposition, processes that serve as powerful metaphors for the transformative and liminal nature of birth. Anthropological and feminist scholars have long described birth as a rite of passage in which the process of transformation is central (36, 37, 60). Similarly, Dahan (12) uses the concept of “birthing consciousness” to capture childbirth not only as a physiological event but as a profound, liminal experience that traverses the boundary between self and other, life and death, dissolution and becoming. In fungal life, decomposition is not an endpoint but a vital stage of renewal. Fungi are nature's master recyclers, breaking down organic material to return nutrients to the ecosystem and enable new life to emerge (16). As Sheldrake (61) notes, this decomposition is less about destruction and more about rearranging possibilities -transforming matter into new forms. This biological process offers a rich metaphor for birth. Just as fungi flourish by turning decay into new life, the birthing subject navigates a state of temporary dissolution that allows for the emergence of new forms of self and relation. Birth, then, is not only about bringing new life but also about the birthing person's reconstitution -a becoming that is contingent on the temporary disintegration of what was (illusorily) perceived as known, stable, and individual.

Fungi offer a compelling framework through which to challenge the foundational assumptions about the neoliberal subject, namely that it is autonomous, self-contained, and in constant pursuit of individual gain. As Sheldrake (16) observes, fungi constitute the living infrastructure through which much of life is relationally woven. Their adaptive success, thriving across millions of years and in the most inhospitable conditions, signals an evolutionary strategy rooted not in competition or sovereignty but in interdependence, flexibility, and symbiosis. Fungi model an ontology in which entanglement is not a liability but a condition for survival, revealing humanity to be irreducibly porous, embedded within ecological, microbial, and material relations. As Anna Tsing (17) writes in her study of matsutake mushrooms, fungi disrupt the fantasy of “alienation—that is, the ability to stand alone, as if the entanglements of living did not matter” (5). They offer a biological and philosophical counterpoint to the competitive logic of neoliberalism, demonstrating that “the important stuff for life happens in collaborations and transformations involving others” (29).

As we have explored in the experiences of birthing individuals like Sara and Michelle, control is not a fixed state but a dynamic interplay among agency, environment, and trust. For some, like Sara, maintaining control was a protective response to a threatening and disempowering context; for others, like Michelle, feeling safe in advance made it possible to relinquish control during labor. This paradoxical nature of control in childbirth, where true surrender can only occur when a sense of safety and agency is first established (13), finds an illuminating analogue in the fungal world. As Sheldrake (61) reflects, engaging with fungi, particularly in fermentation, reveals that control is never absolute but instead always relational. One can set certain parameters, such as adjusting the temperature and managing the oxygen, but the microbial cultures will respond in their own ways. Thus, rather than commanding outcomes, the fermenter is entering into a dance with wild populations of fungi and bacteria, learning to guide while also letting go. This mirrors what many birthing individuals describe: on the one hand the need to prepare, to assert preferences, to navigate the systems that often undermine autonomy (62, 63) but also, on the other hand, a kind of surrender that the process demands once labor begins and that is only possible when the conditions for safety and respect have already been established (3, 12). It is this relational mode of being, rather than individual mastery, that allows both fermentation and birth to unfold as creative, transformative processes. In both cases, control is not something to wield but something to recalibrate, a fluid balance between structure and openness, boundaries and surrender. Learning from fungi, we begin to understand that not being fully in control does not mean chaos—it can mean collaboration with life.

There is also another way in which fungi challenge the logic of control. Consider the technocratic model of childbirth, where birthing women are stripped of their individuality and reduced to machine-like bodies on an industrial assembly line (32, 36, 37). Drawing on Anna Tsing's (17, 64) analysis, we can liken this model to the structure of colonial-era plantations, which were deliberately organized around the principle of alienation in order to maximize control. These plantations introduced monocultures—non-native crops planted on land cleared of local vegetation—that were tended by enslaved laborers who had been forcibly detached from their communities. As Tsing (64) notes, plantation agriculture sought superabundance through the domination of a single crop. “But one ingredient [was] missing: They remove[d] the love” (148). In other words, they removed the element of care and relational connection. “Instead of the romance connecting people, plants, and places, European planters introduced cultivation through coercion” (148). Many fungi, in stark contrast—such as the matsutake mushroom that Tsing (17) focuses on and that “cannot live without transformative relations with other species” (40)—resist such industrial domestication. They flourish only through mutualistic, multispecies relationships, nourished by trees and thriving in the dynamic entanglements of the forest. This “contaminating relationality” (40), which embodies a form of life that defies hierarchical, alienating logics, makes species like the matsutake unfit for cultivation within monocrop systems. Similarly, human childbirth depends on the vital elements of love and connection, and it may be compromised when it is situated within systems structured by alienation instead. We birth with others, and as such, fostering the essential interdependence and relationality that underpin childbirth is vital to ensuring positive experiences and resisting the alienation that threatens them (4).

In order to help to grasp the value of the fungal turn as a lens on childbirth, Table 1 outlines key synergies and their practical implications for fostering positive childbirth environments. Although such implications are well reflected in existing world guidelines advocating for respectful childbirth care, their comprehensive adoption remains insufficient (65). The dominant technocratic model, emphasizing standardized protocols and medical intervention, continues to prevail in many obstetric contexts, thereby impeding the consistent application of humanistic and relational approaches that foster positive childbirth experiences.

**Table 1:** Fungal principles applied to childbirth: implications for obstetric care.

Fungal principle	Birth analogy	Implications: what does this mean in obstetric care?
Mycelial networks: Vast, interconnected webs sustain fungal life through mutual support and resource sharing.	Childbirth unfolds within dense relational networks -between birthing person, baby, caregivers, communities, and environments.	Positive birth thrives in collective, supportive environments rather than isolation. Collaborative care models involving doulas, midwives, family, and community are encouraged.
Porosity and permeability: Fungi exist as interconnected organisms, sharing nutrients and genetic material, with blurred boundaries between individuals.	Pregnancy and childbirth are not isolated events but part of a new, multi-species community that transcends individual boundaries.	Trust and care allow surrendering rigid control. Care practices and environments that foster trust, safety, and fluid support, respecting and engaging fully with the birthing process.
Transformation through decomposition: Fungi use decay as a vital process for renewal and new growth.	Birth involves a liminal, transformative dissolution of previous selfhood, enabling emergence of new identities and relations.	Recognizing birth as liminal transformation reframes it as more than a medical event -it's an ontological shift. Support birthing people through the emotional and physical transformation of birth by integrating trauma-informed care and reflective practices into obstetric care.
Resistance to monocultures: some fungi require multispecies relationships and cannot be industrially domesticated.	Childbirth flourishes when love, care, and relational connection are central -not when reduced to a standardized, alienating procedure.	Care models that move away from rigid, protocol-driven birth practices towards flexible, individualized care that respects birthing person's preferences and relational needs.
Relational control: Fungal processes like fermentation require guiding parameters but allow for unpredictable collaboration.	Birth balances preparation and agency with surrender to the process, within a relational dynamic.	Guidance is fluid and collaborative. Thus, a medical oversight should be balanced with respect for individual needs and rhythms during childbirth, emphasizing supportive guidance rather than strict control or intervention.

## Conclusions

The fungal logic has profound implications for how we understand birth. The modern medicalization of childbirth, marked by control, standardization, and risk management, can be read as a symptom of a broader cultural alienation: from our bodies, from ecological temporality, and from collective modes of care. Science is increasingly questioning rigidly individualistic models of life and moving toward more ecological frameworks; fungi embody this turn. Reframing childbirth through a fungal lens allows us to see it as a deeply ecological and collaborative process.

It is of course not impossible to have a positive birth experience in scenarios where women are suspicious about the degree to which they will be protected and must therefore find solace in exercising their autonomy and control and protecting themselves from coercion or violence. However, this is a very low bar for birth experiences, and it usually puts too much responsibility on the birthing woman, frequently producing strong feelings of self-blame if she does not get to experience the birth that she expected and hoped for. A higher and more appropriate standard for birth experiences is one that begins with a setting in which the birthing subject feels cared for and protected—where she is not required to defend herself or constantly protect her body from unwanted intervention. Births experienced under such conditions have been reported as deeply rewarding and as allowing expansiveness, flow, and sometimes profound transformation. In a protected space where the birthing subject can let itself go into a fluid state of being, blurring its boundaries and experiencing intercorporeality and interconnectedness, the “person” does not need to maintain the traditional boundaries that are perceived as separating her from the world and from others, protecting her, among other things, from others' intrusions (12, 14). Under these optimal conditions, the person is transformed into a kind of fungus, a porous being, a less solid and much spongier, waterier one, who can birth not through autonomy and agency but through expansiveness, fluidity, and a blurred, leaky, permeable intertwining with her surroundings.

Like fungal networks, birth is an entangled transformation—one that calls for relationality, adaptability, and respect for porous boundaries. Seen in this light, it is not only politically and ethically urgent but ecologically essential to move toward models of birth that prioritize connection over control. As Tsing (17) reminds us, “survival requires livable collaborations” (29)—a truth that fungi have long embodied and one by which we, too, might yet learn to live.

## Data Availability

The original contributions presented in the study are included in the article, further inquiries can be directed to the corresponding author.

## Footnotes

1 In the context of positive birth, autonomy is many times experienced simply as “control” or as a sense of making decisions sovereignly, without coercion and “freely”. However, autonomy in such context can also appear as ongoingly created through relations and interconnectedness, that is, through an intertwining with significant others surrounding or accompanying the birthing person (among them also her still-in-womb baby). Such “relational autonomy” has been thoroughly discussed within recent feminist and care ethics [see for instance: (4–6)]. These two diverse ways of conceiving autonomy shall inform our following discussion on different forms of experiencing a “good birth”.

2 We emphasize that experiences of transcendence, such as those described by Michelle, may or may not occur in physiological, unmedicated births. We do not wish to essentialize birthing experiences or suggest, in any way, that decisions regarding medication or interventions are inherently better or worse. Each experience is deeply personal and unique, shaped by women's diverse life histories and needs; as such, care should be respectful, individualized, and tailored accordingly.
